# Role Played by Signalling Pathways in Overcoming BRAF Inhibitor Resistance in Melanoma

**DOI:** 10.3390/ijms18071527

**Published:** 2017-07-14

**Authors:** Xian Yang Chan, Alamdeep Singh, Narin Osman, Terrence J. Piva

**Affiliations:** 1 School of Health & Biomedical Sciences, RMIT University, Bundoora 3083, Victoria, Australia; s3321036@student.rmit.edu.au (X.Y.C.); s3543793@student.rmit.edu.au (A.S.); narin.osman@rmit.edu.au (N.O.); 2 Department of Immunology, Monash University, Melbourne 3004, Victoria, Australia; 3 Department of Pharmacy, University of Queensland, Woolloongabba 4102, Queensland, Australia

**Keywords:** melanoma, cell signalling, BRAF, MAPK, RTK, PI3K-AKT-mTOR

## Abstract

The discovery of the BRAF^V600E^ mutation led to the development of vemurafenib (PLX4032), a selective BRAF inhibitor specific to the kinase, for the treatment of metastatic melanomas. However, initial success of the drug was dampened by the development of acquired resistance. Melanoma was shown to relapse in patients following treatment with vemurafenib which eventually led to patients’ deaths. It has been proposed that mechanisms of resistance can be due to (1) reactivation of the mitogen-activated protein kinase (MAPK) signalling pathway via secondary mutations, amplification or activation of target kinase(s), (2) the bypass of oncogenic pathway via activation of alternative signalling pathways, (3) other uncharacterized mechanisms. Studies showed that receptor tyrosine kinases (RTK) such as PDGFRβ, IGF1R, EGFR and c-Met were overexpressed in melanoma cells. Along with increased secretion of growth factors such as HGF and TGF-α, this will trigger intracellular signalling cascades. This review discusses the role MAPK and Phosphatidylinositol-3-kinase-protein kinase B-mammalian target of rapamycin (PI3K-AKT-mTOR) pathways play in the mechanism of resistance of melanomas.

## 1. Introduction

There are three main types of skin cancer: melanoma, squamous cell carcinoma (SCC) and basal cell carcinoma (BCC), the latter two are collectively known as non-melanoma skin cancers (NMSC). NMSC are derived from keratinocytes, whereas melanomas are derived from melanocytes [1]. Although NMSC are more predominant than melanoma, they are less aggressive and rarely metastasize except for some SCCs [2]. Melanomas are highly metastatic and secondary tumours are often observed in the lung, liver and brain. Australia has the highest incidence of melanoma in the world, with more than 12,700 cases reported in 2013 and the number is expected to escalate to 14,000 in 2017. Melanoma is the third most common cancer in Australia, accounting for more than 1500 patient deaths each year [3]. Despite being the least common type of skin cancer (2% of all skin cancers), it has the highest fatality rate, accounting for 75% of all skin cancer deaths [3].

If detected early, non-metastatic melanoma can be treated with surgery [4] or chemotherapy using dacarbazine [5,6]. Unfortunately, the survival rates for dacarbazine treatments are ~10% [5]. However, the challenge lies in treating metastatic melanoma as currently there are no treatment(s) that can significantly improve survival time or rates. Recent analysis of the melanoma genome has shown that these metastatic tumours can be subdivided into four subtypes, based on their mutation profile: BRAF (v-raf murine sarcoma viral oncogene)-driven (~52%), NRAS (N-Rat sarcoma)-driven (~28%), NF1 mutated (14%) and the rest being classified “triple wild-type” [7,8]. With the exception of the latter subtype, all these melanomas possess mutations that affect signalling through the MAPK pathway. Considerable effort has been directed at understanding the effect these mutations have on the activity of intracellular signalling pathways and how they interact to overcome the effects of specific inhibitors. In the case of patients who have BRAF-driven mutations, on 17 August 2011, the U S Food and Drug Administration (FDA) approved a new drug, vemurafenib (PLX4032), a selective BRAF inhibitor (BRAFi) for the treatment of advanced metastatic melanoma [5,9,10]. For a short period following treatment, the melanomas regressed and patients had an improved quality of life; however, these tumours become resistant to vemurafenib, eventually resulting in their deaths [11,12]. Resistance to BRAFi has predominantly been shown to be related to the reactivation of the MAPK signalling pathway (BRAF-MEK-ERK–BRAF-MAPK/ERK kinase-extracellular signal-regulated kinase), however, other mechanisms e.g., upregulation of PI3K-AKT-mTOR signalling, increased expression of growth factor receptors on the cell membrane have been shown to be involved [13,14]. It was reported by Rizos et al. [15] that up to 40% of melanoma patients had unidentified mechanisms of resistance.

## 2. The BRAF-MEK-ERK Pathway

BRAF is a serine-threonine protein kinase that is responsible for signal transduction inside cells, directing normal cell growth, proliferation, differentiation and survival [5]. Similar to its other isoforms—ARAF and CRAF/RAF-1—BRAF is activated by upstream RAS, which will in turn phosphorylate MEK (MAPK/ERK kinase), leading to the activation of ERK (extracellular signal-regulated kinase) pathway [5,16,17]. Phosphorylated ERK1/2 then activates transcription factors Elk 1, c-Fos and c-Myc, giving rise to normal cell growth, proliferation, differentiation, migration, angiogenesis and survival [16,17].

Davies et al. [18] first observed that BRAF mutations were prevalent in ~50% of melanomas. The most common is a point mutation at nucleotide 1799 in which valine is substituted with glutamic acid at codon 600; this gives rise to the term BRAF^V600E^ [18]. Other BRAF mutations (V600K, V600D and V600R) exist, but the prevalent (90%) form is V600E [19]. Melanomas harbouring this V600E mutation have higher kinase activity than BRAF^WT^ (WT denotes wild-type) [9]. As a result, this oncogenic BRAF^V600E^ is constitutively activated independent of its upstream activator protein-RAS, resulting in increased stimulation of its downstream effector proteins-MEK and ERK via phosphorylation. This leads to melanoma cell survival, proliferation, tumour angiogenesis and metastasis via the ERK signalling pathway [10,17,18]. The development of a specific BRAF^V600E^ inhibitor has highlighted this kinase as an important molecular target in melanoma therapy. Although this BRAF mutation can play a significant role in melanoma development, it is not responsible for the development of these tumours [20]. Other mutations are needed for melanocytes to become cancerous; most notable are ultraviolet (UV)-induced mutations, which include NRAS, p16, p53 and PTEN (Phosphatase and tensin homolog) mutations [21,22]. It should be noted that BRAF^V600E^ is not a ultraviolet (UV) signature mutation [23]. UVB and UVA mutations observed in melanoma are mainly characterized by C→T and G→T transitions, respectively [24,25]. UV-induced PTEN mutations are observed in exons 2 and 6, while germline mutations mainly occur in exon 5; in some cases this protein is deleted, resulting in a loss of its function in 20–40% melanoma cells [8,13,14,26]. Other mutations observed in MAPK signalling intermediates in melanoma cells include MEK1 (C121S and P124L), MEK2 (Q60P and C125S) and AKT1 (E17K) [13,27,28].

**Figure 1:**
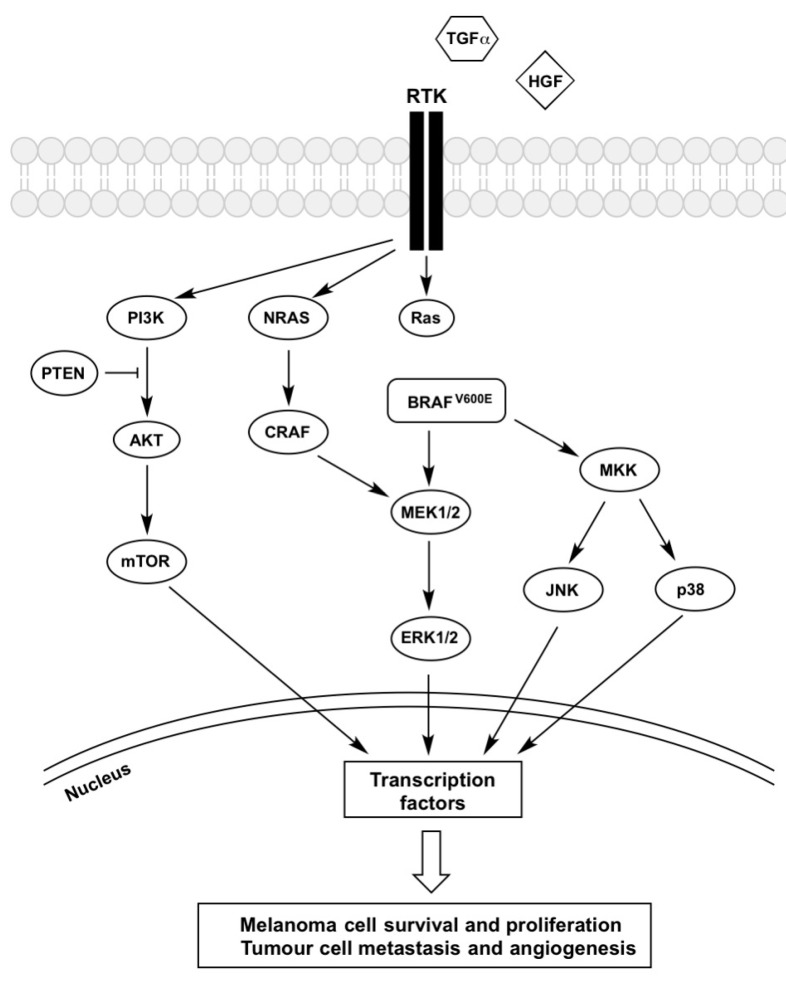
Mechanisms of BRAFi resistance converge on constitutive ERK signalling. Regardless of its upstream activator RAS, BRAF^V600E^ is constantly activated, and vemurafenib treatment was shown to inhibit the kinase. However, the increased secretion of growth factors (HGF and TGF-α) coupled with the overexpression of receptors (PDGFRβ, IGFR1, EGFR and c-Met) trigger the activation of signalling cascades inside melanoma cells that bypasses BRAFV^600E^ inhibition, which confers resistance to these therapeutic agents. Both MAPK and PI3K-AKT-mTOR pathways are activated and the signal is passed into the nucleus, which triggers phosphorylation of transcription factors, leading to melanoma cells survival and proliferation as well as tumour metastasis and angiogenesis. Description of the abbreviations listed above are contained within this review.

Vemurafenib is a highly potent ATP-competitive inhibitor of BRAF^V600E^ and it has been shown to interrupt the BRAF-MEK-ERK signalling pathway (Figure 1). It causes a decrease in ERK activation in BRAF^V600E^ melanoma cells, resulting in G1 phase cell cycle arrest, inducing cell death [29,30,31]. Vemurafenib has been shown to reduce the abundance of anti-apoptotic proteins [32] as well as increase that of the pro-apoptotic proteins PUMA (p53 upregulated modulator of apoptosis) and BIM (Bcl-2-like protein 11) [33]. This imbalance resulted in an increase in cytosolic Ca^2+^ levels which induced endoplasmic reticulum (ER) stress-mediated apoptosis in BRAF^V600E^ melanoma cells [32]. Hatzivassiliou et al. [29] and Poulikakos et al. [30] both observed that vemurafenib did not inhibit wild-type BRAF, but instead paradoxically stimulated its activity, which increased tumour growth. It is believed that this is due to the transactivation of BRAF^WT^ and CRAF homo- or heterodimers.

### BRAF Inhibitor Resistance

Unfortunately, the relative initial success of vemurafenib has been dampened by the development of acquired resistance to the drug [12,17]. Wagle et al. [12] first described resistance to BRAF inhibition in a 38-year old male melanoma patient. Initial treatment with vemurafenib caused complete regression of all subcutaneous tumour nodules within 15 weeks. However, after 16 weeks of treatment, the patient experienced widespread melanoma relapse, rapid disease progression and, after 23 weeks, had died. The survival rates of vemurafenib-treated melanoma patients are not significantly better to that observed in untreated patients. In general, mechanism of therapeutic resistance in kinase-driven cancers can be due to (1) reactivation of the mitogen-activated protein kinase (MAPK) signalling pathway via secondary mutations, amplification or activation of target kinase(s), (2) the bypass of oncogenic pathway via activation of alternative signalling pathways, (3) other uncharacterized mechanisms. Resistance to BRAF inhibitor (BRAFi) can occur either upstream or downstream of this kinase, and has also been shown to be either ERK-dependent or -independent [12,34,35,36].

In ERK-dependent resistance, it has been proposed that the upstream activator protein of BRAF-NRAS is mutated (~20% of NRAS is mutated in melanomas, the most common ones being Q61R/K/L) and bypasses BRAF inhibition via ARAF and/or CRAF, resulting in the reactivation of MAPK signalling pathways [12,35,36,37] as well as that of the PI3K-AKT-mTOR (phosphatidylinositol-3-kinase-protein kinase B-mammalian target of rapamycin) pathway [38]. Sanchez-Laorden et al. [39] discovered that BRAFi were shown to stimulate metastasis in RAS mutant or inhibitor-resistant cultured (human and mice) melanoma cells through the reactivation of ERK signalling pathways. In general, RAS stimulates interleukin-8 (IL-8) expression via activation of the activating protein-1 (AP-1) transcription factor by the RAF-ERK and RAC (RAS-related C3 botulinum toxin substrate)-JNK (c-Jun N-terminal kinase) pathways as well as through the activation of NFκB (nuclear factor kappa-light-chain-enhancer of activated B cells), resulting in protease-dependent mesenchymal invasion and metastasis [39]. However, in the presence of oncogenic RAS, BRAF inhibitors hyperactivate this pathway, leading to increased expression of IL-8, uPA (urokinase-like plasminogen activator) and MMP-1 (matrix metalloprotease-1) proteins [39]. Therefore, once BRAF-mutant melanoma cells become resistant to BRAF inhibitors, not only do the cells evade inhibitory effects of these drugs, but their ability to metastasize was also enhanced. This result further supported the earlier finding of Wagle et al. [12] who observed that when melanomas relapse, they were often much more aggressive and had higher rates of progression. The alternative spliced variant form of BRAF^V600E^ in some resistant melanoma cells could also render vemurafenib ineffective to ERK signalling inhibition [40]. Several findings also suggested that the PI3K-AKT-mTOR signalling pathway has a role to play in BRAFi resistance development, triggering an alternative survival pathway by decreasing apoptosis occurring in these cells [17,41,42].

In contrast, in ERK-independent resistance, platelet-derived growth factor receptor-β (PDGFRβ) is overexpressed in BRAF^V600E^ inhibitor-resistant melanoma cells, leading to renewed proliferation and tumour growth [34,37]. Nazarian et al. [37] observed that by silencing PDGFRβ expression in BRAF^V600E^-inhibited melanoma cells, restoration of the apoptotic pathways in these cells did not occur, indicating that the upregulation of this growth factor receptor was not the only mechanism of resistance. Indeed, the increased activation of insulin-like growth factor 1 receptor (IGF1R) by IGF1 can activate the RAF-MEK-ERK and/or PI3K-AKT pathways and may play a role in the development of melanoma resistance [34,43]. Villanueva et al. [43] showed that IGF1R was overexpressed in resistant melanoma cells and combined inhibition of IGF1R- and MEK-induced dramatic apoptosis, which suggested that ERK-independent resistance was mediated by IGF1R signalling in BRAFi-resistant cells, as a result of the activation of the PI3K-AKT signalling pathway. Another BRAF inhibitor resistance mechanism is based on the tumour microenvironment. Recently, Straussman et al. [44] showed that hepatocyte growth factor (HGF) secreted by the surrounding stromal fibroblasts activated the HGF receptor c-Met, resulting in the reactivation of the MAPK and PI3K-AKT-mTOR signalling pathways in BRAFi-resistant melanoma cells. HGF-expressing stromal cells appeared following treatment with BRAF^V600E^ inhibitors in a melanoma patient [44].

The discovery of constitutive activity of the BRAF-MEK-ERK signalling pathway in melanomagenesis emphasizes the importance of these signalling pathway intermediates as potential therapeutic targets in treating melanoma especially those that have become resistant to BRAF^V600E^ inhibitors. Regardless of the mechanism(s), the final cause of resistance seems to converge on constitutive activation of ERK signalling. However, due to melanomas multifactorial nature, it is also important to examine and understand the involvement of other cell signalling pathways that may play a role in this process.

## 3. Receptor Tyrosine Kinases (RTK)

The majority of MAPK cell signalling pathways originate from RTK activation. RTKs are cell surface receptors which receive extracellular stimuli and then relay the message into the cells to trigger signalling cascades, mediating cellular processes including cell proliferation, differentiation, survival and migration [45]. As noted above, the discovery by Straussman et al. [44] as well as the increased expression level of PDFGRβ [37] and IGF1R [43] in melanoma cells, which led to the development of resistance, has spurred interest in the role of growth factors and their receptors in BRAFi resistance.

Girotti et al. [46] discovered that the number of epidermal growth factor receptors (EGFR) were upregulated in BRAFi-resistant melanoma cell lines. The increased phosphorylation of EGFR in these cells not only promoted proliferation, but also drove invasion and metastasis via the EGFR-SRC family kinase (SFK)-signal transducers and activators of transcription 3 (STAT3) pathway. It was suggested that when melanoma cells became resistant, this pathway is upregulated. However, the treatment with EGFR inhibitor along with BRAFi successfully blocked the growth of these resistant cells [46]. Another study also showed that EGFR is upregulated in both BRAFi-resistant melanoma cell lines and patient tumours [47]. However, the overexpression is due to epigenetic regulation rather than activation of the EGFR-SFK-STAT3 pathway, and as a result, the PI3K-AKT-mTOR pathway was hyperactivated. Similar to the study by Girotti et al., treatment with EGFR and BRAF inhibitors was proved to be effective in overcoming BRAFi resistance [46]. Apart from EGF, transforming growth factor-α (TGF-α) can also bind to EGFR [48]. TGF-α is a powerful mitogenic growth factor found in the skin. Its expression in the skin is upregulated after exposure to UV radiation [49], most likely due to increased cell surface protease activity as seen in UV-irradiated cells [50]. Elevated TGF-α levels induce changes in gene expression which can result in uncontrolled growth and avoidance of apoptosis in UV-irradiated skin cells [51]. Although high levels of TGF-α along with TNF-RII (tumour necrosis factor α receptor II), TIMP-1 (tissue inhibitor of metalloproteinases 1) and CRP (C-reactive protein) correlates with poor prognosis [52], the role played by TGF-α in melanomagenesis is currently not well understood. However, it was reported that TGF-α promoted osteosarcoma metastasis by inducing ICAM-1 (intercellular adhesion molecule-1) and activated the PI3K-AKT signalling pathway and eradication of these two, either by siRNA or specific inhibitors, proved to be effective in reducing TGF-α-stimulated osteosarcoma cell migration [53].

Furthermore, the c-Met receptor to which HGF binds exclusively is expressed and activated in melanoma cells and tumours, especially in those that harbour the NRAS mutation and non-mutated BRAF [54]. Activation of the c-Met receptor promotes growth and proliferation in BRAFi-resistant melanoma cells [44] as well as metastasis [55,56]. Vice versa, inhibition of the receptor halted the growth of melanoma cells by inducing apoptosis and differentiation [55], but the efficacy of the c-Met receptor inhibitor was limited due to the development of resistance [57]. This leads to an investigation of combination therapy. Etnyre et al. [58] demonstrated that treatment of c-Met receptor inhibitor together with a BRAFi was effective in BRAF^V600E^ melanoma cells. They also highlighted the importance of PI3K-AKT-mTOR and Wnt (Wingless-related integration site) signalling pathways in c-Met inhibitor resistance, and the inhibition of all the targets mentioned was proved to be effective in overcoming this resistance. Vergani et al. [59] showed that combination treatment of vemurafenib with siRNA targeting the c-Met receptor was effective in reversing the effects of HGF-induced cell growth as well as inhibiting melanoma cell invasion and metastasis. Cao et al. [56] also showed that the migration and invasion of HGF-stimulated melanoma was inhibited by quercetin, a dietary flavonoid. Quercetin treatment decreased phosphorylation and dimerisation of c-Met receptors, which in turn reduced the phosphorylation of Gab1 (Grb2-associated binding protein 1), resulting in the inhibition of FAK (focal adhesion kinase) and PAK (p21-activated kinase) activation, which are critical in cell migration and invasion [56]. Overexpression of RTKs coupled with the increased secretion of their ligands activates the corresponding downstream signalling cascade(s), enabling proliferation, growth and metastasis of melanoma cells regardless of their drug resistance status.

## 4. PI3K-AKT-mTOR Pathway

**Figure 2:**
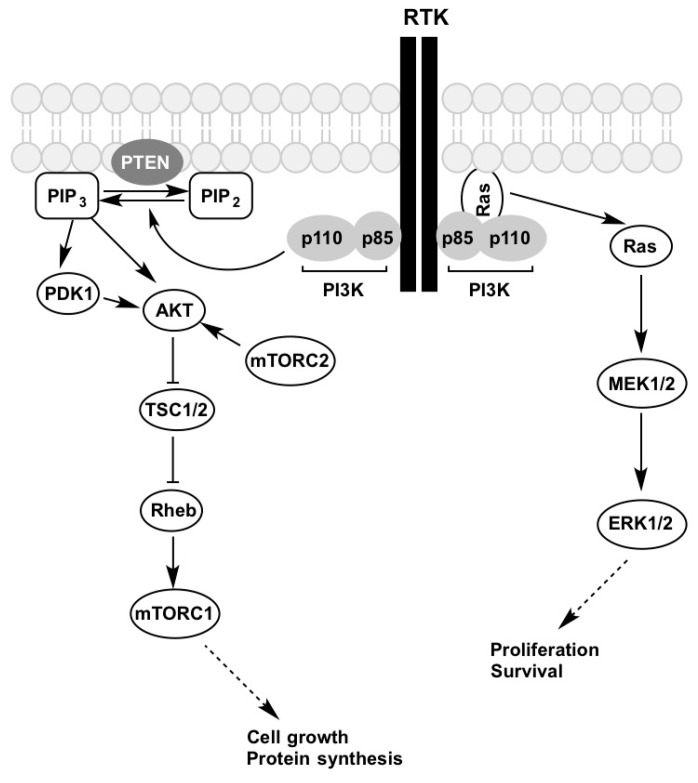
Growth factor activation of the PI3K-AKT-mTOR and MAPK signalling pathways. Figure is adapted from [67]. Description of the abbreviations listed above are contained within this review.

Apart from RAS-RAF-MEK-ERK pathway, the PI3K-AKT-mTOR pathway is involved in regulating normal cell growth, proliferation, differentiation as well as tumorigenesis [17,44,60,61]. Growth factor activation of RTK results in PI3K binding through its p85 regulatory subunit to the phosphorylated adaptor proteins on the cytoplasmic region of these receptors (Figure 2). This results in the release of the p110 catalytic subunit which then phosphorylates phosphatidylinositol bisphosphate (PI(4,5)P_2_) to phosphatidylinositol triphosphate (PI(3,4,5)P_3_). PTEN causes the reversal of PIP_3_ to PIP_2_; this is considered to be its tumour suppressor activity as it inhibits the downstream phosphorylation of AKT [62]. The loss of PTEN function has been implicated in the development of many different types of cancer [63] including melanoma [13,26,64]. PIP_3_ recruits 3-phosphoinositide-dependent protein kinase-1 (PDK1) and AKT to the cell membrane. Liu et al. (2015) found that PIP_3_ binds to mTORC2 which releases the mTOR kinase from its autoinhibitory activity [65]. AKT possesses two phosphorylation sites: Ser473 and Thr308. Thr308 is located in the activation loop of the catalytic protein kinase core and is phosphorylated by PDK1, while Ser473 is located in a C-terminal hydrophobic motif and is phosphorylated by mTOR complex 2 (mTORC2) [66]. Phosphorylation of AKT at these two sites results in its activation, which causes downstream inhibition of tuberous sclerosis complex 1 and 2 (TSC1/2). TSC1/2 usually converts Ras homolog enriched in brain (Rheb-GTP) to Rheb-GDP, and, if inhibited, this results in the accumulation of the former in the cell. Rheb-GTP in turn activates mTORC1, which promotes cell growth and protein synthesis [62].

In those melanomas, which possess PTEN mutations, the PI3K-AKT-mTOR pathway becomes hyperactive [21,22], contributing to tumour progression. Apart from PTEN mutations/deletions, AKT3 has also shown to be activated in 40–60% of melanomas, which stimulates the activity of this pathway [28,68]. It was suggested by Tumaneng et al. [69] that PTEN regulation of PI3K pathway involved the Hippo-Yes associated protein (YAP) pathway. YAP, a downstream effector of Hippo, mediates crosstalk between Hippo and the PI3K-AKT-mTOR pathway via suppression of miR-29, which inhibits PTEN. The activity of PI3K-AKT-mTOR pathway was also shown to be upregulated as a result of the overexpression or hyperactivation of RTKs such as c-Met, EGFR and IGF1R in melanoma cells [43,44,46,47]. Together with the constitutive activation of ERK signalling, this can lead to the proliferation and survival of melanoma cells. Recent studies have shown that the PI3K-AKT-mTOR pathway plays a role in the development of BRAFi resistance by triggering an alternative signalling survival pathway, leading to decreased levels of apoptosis in melanoma cells [45,70].

Deuker et al. (2015) found that the use of inhibitors against the α, δ and γ isoforms of the p110 catalytic subunit of PI3K in conjunction with BRAF inhibitors resulted not only in a greater rate of tumour regression in melanoma cells, but the combination treatment also forestalled the onset of MEK inhibitor resistance [71]. Recently, Meierjohann (2017) suggested that crosstalk between the MAPK and PI3K/AKT/mTOR pathways may be involved in the development of treatment resistance in melanoma [72]. A consequence of downstream BRAF signalling is the production of Sprouty (SPRY) and Sprouty-related EVH1-containing domain (SPRED) family members, which negatively regulate RAS activity. As a result of vemurafenib inhibition of BRAF^V600E^, levels of SPRY and SPRED family members are reduced, thereby resulting in the activation of both RAS and PI3K/AKT signalling activity. PI3K activity has been shown to protect melanoma cells from genotoxic stress by suppressing the apoptosis mediator PUMA [72]. The increase in RAS activity due to the reduction in SPRY2 levels in melanoma cells resulted in the activation of CRAF signalling overcoming the inhibition of BRAF^V600E^ by vemurafenib [73].

Crosstalk between the PI3K and MAPK pathways has been observed in other cancers. For example, Will et al. [74] showed that induction of apoptosis in BT-474 breast cancer cells by PI3K inhibitors was dependent upon inhibition of the RAS-RAF-MEK-ERK signalling pathway. The PI3K inhibitors caused a rapid decline both in AKT and RAS wild-type tumours, suggesting that these two pathways are connected to each other and PI3K functions upstream of RAS to induce rapid apoptosis. Cheung et al. [75] demonstrated that the PIK3R1^R348^ (phosphatidylinositol 3-kinase regulatory subunit α) and PIK3R1^L370fs^ gene mutation in p85α regulatory subunit of PI3K render BaF3 endometrial cancer cells sensitive to MAPK signalling inhibitors as well as induce ERK and JNK activation. These findings underlie the crosstalk that occurs between the PI3K and MAPK pathways and, together, they are able to promote proliferation and survival of melanoma cells.

## 5. JNK Pathway

The JNKs are stress-activated serine-threonine protein kinases that also belong to the MAPK pathway [76]. They were originally identified by their ability to phosphorylate c-Jun in response to UV-irradiation, but now are recognized as important regulators of cell proliferation, survival, death, DNA repair and metabolism [76,77]. Although they are activated in a similar fashion to ERK, they have different upstream activator proteins. Instead of RAF, RAS activates RAC (MAPKKK), which in turn activates MKK4/7 (MAPKK) and then JNK1/2/3 [77]. When activated, the JNKs translocate into the nucleus to activate transcription factors, triggering cellular responses. In the presence of interleukin-1α (IL-1α), the JNK pathway has been shown to be involved in UV-induced TNF-α release in keratinocytes, but the mechanism involved is not clear [78,79]. Karin et al. [76] observed that a modest and transient JNK activation promoted TNF-α-induced cell proliferation, whereas, the opposite could lead to TNF-α-induced cell death. According to Alexaki et al. [80], the levels of p-JNK1 and p-JNK2 were highly variable across melanoma cell lines, and the former isoform was responsible in promoting cell growth. Inhibition of JNK by SP600125 successfully attenuated growth of melanoma cells either by inducing G2/M cell cycle arrest or triggering apoptosis.

## 6. p38 MAPK Pathway

The p38 MAPK pathway is activated in a similar fashion to that of ERK, but instead of MEK, MKK (MAP kinase kinase) can also be activated by RAF, which in turn phosphorylates p38 [77]. Similar to JNK, it can be activated by stimuli such as pro-inflammatory cytokines and environmental stresses (e.g., UV radiation), and is thought to play a role in cell survival, death, differentiation, apoptosis, cell-cycle checkpoints, drug resistance, migration and metastasis [81]. Like the JNK pathway, p38 MAPK pathway plays a role in UV-induced TNF-α release in both melanocytes and keratinocytes treated with IL-1α [78,79]. These authors noted that IL-1α did not stimulate the secretion of TNF-α from UV-irradiated melanoma cells. Furthermore, p38 MAPK pathway has been shown to be involved in pro- and anti-apoptotic events in tumours as well as in inflammatory and tumorigenic responses [81]. Keuling et al. [82] demonstrated that combination treatment of p38 inhibitor (SB202190) and Bcl-2 family inhibitor (ABT-737) synergistically induced apoptosis in melanoma cells. Although melanoma cell viability was decreased in response to p38 inhibitor treatment, this was due to growth arrest rather than the induction of apoptosis [82]. These findings suggest that the p38 MAPK pathway could be activated when BRAF is inhibited in melanoma cells.

## 7. Conclusions

In summary, due to its multifactorial nature, understanding the mechanism of BRAFi resistance is one of the greatest challenges in melanoma research. Growth factors allow BRAF-inhibited melanoma cells to overcome inhibition by activating alternative signalling pathways. MAPK, as well as the PI3K-AKT-mTOR pathway, also play a role in the proliferation and survival of melanoma cells. Since BRAF signalling belongs to the MAPK signalling pathway and PI3K-AKT-mTOR pathway can provide an alternative survival pathway for melanoma cells, it is important to understand which pathway(s) are activated by growth factors when BRAF^V600E^ is inhibited to gain further insights about the mechanism of BRAF resistance. Armed with such information, it will assist in the development of more effective treatment regimes. Current studies involve combination therapies to target multiple signalling pathways for the treatment of metastatic melanoma as evidenced by the FDA-approved combination therapy of dabrafenib and trametinib for the treatment of patients with BRAF^V600E/K^-mutant metastatic melanoma in January 2014 [83]. Due to its multifactorial nature and the ineffectiveness of targeted therapy, immunotherapy such as Ipilimumab, interleukin-2 and PD-1 (programmed death-1) receptor antagonist were developed [84]. For more information on current immunotherapeutic interventions in treating melanomas, please see [85,86]. However, the involvement of other players such as G-protein-coupled receptor (GPCR) signalling as well as the tumour microenvironment might also have a significant role to play in allowing BRAFi-resistant melanoma cells to overcome current treatment strategies. The challenge is to identify the role these signalling pathways play and to devise treatment strategies that may successfully eradicate these melanomas.

## Abbreviations

BIM	Bcl-2-like protein
BRAF	v-Raf murine sarcoma viral oncogene homolog B
BRAFi	BRAF inhibitor
EGFR	Epidermal growth factor receptor
ERK	Extracellular signal-regulated kinase
HGF	Hepatocyte growth factor
IGF1R	Insulin growth factor 1 receptor
JNK	c-Jun N-terminal kinase
MAPK	Mitogen-activated protein kinase
MEK	MAPK/ERK kinase
mTORC	mammalian target of rapamycin (mTOR) complex
PDGFRβ	Platelet-derived growth factor receptor β
PDK1	3-phoshoinositide dependent protein kinase-1
PI3K-AKT-mTOR	Phosphatidylinositol-3-kinase-protein kinase B-mammalian target of rapamycin
PTEN	Phosphatase and tensin homolog
PUMA	p53 upregulated modulator of apoptosis
RAS	Rat sarcoma
RTK	Receptor tyrosine kinase
TGF-α	Transforming growth factor-α
UV	ultraviolet
